# 1,1,2,2-Tetra­kis(1,3-benzoxazol-2-yl)ethene

**DOI:** 10.1107/S1600536811026924

**Published:** 2011-07-13

**Authors:** Tesfamariam K. Hagos, Stefan D. Nogai, Liliana Dobrzańska, Stephanie Cronje, Helgard G. Raubenheimer

**Affiliations:** a Department of Chemistry, University of Stellenbosch, Private Bag X1, Matieland, South Africa; b Department of Chemistry, Katholieke Universiteit Leuven, Celestijnenlaan 200F - bus 2404, B-3001 Heverlee, Belgium; c Institut für Anorganische und Analytische Chemie, Goethe-Universität Frankfurt, Max-von-Laue-Strasse 7, D-60348 Frankfurt am Main, Germany

## Abstract

The title compound, C_30_H_16_N_4_O_4_, reveals 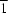 crystallographic and mol­ecular symmetry and accordingly the asymmetric unit comprises one half-mol­ecule. The dihedral angle between the planes of the two geminal benzoxazole rings is 74.39 (5)°. The packing features weak C—H⋯N and π–π inter­actions [centroid–centroid distance = 3.652 (1) Å].

## Related literature

For the chloro­form disolvate of 1,1,2,2-tetra­kis­(1,3-benzo­thia­zol-2-yl)ethene, see: Hagos *et al.* (2010 ▶). For bond lengths in the benzoxazole moiety in related compounds, see: Jian *et al.* (2007 ▶); Lokaj *et al.* (1997 ▶); Muir *et al.* (1992 ▶). For details of the cut-off applied for C—H⋯N inter­actions, see: Desiraju & Steiner (2006 ▶). For the synthesis of AuCl(PPh_3_), see: Bruce *et al.* (1989 ▶).
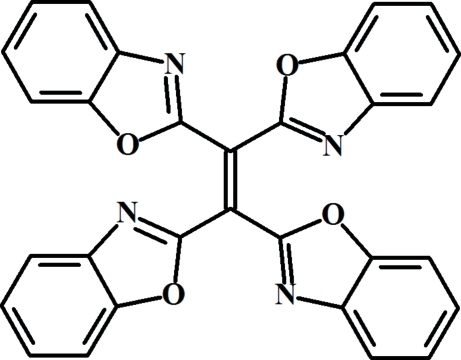


## Experimental

### 

#### Crystal data

C_30_H_16_N_4_O_4_
*M* *_r_* = 496.47
Monoclinic, 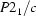

*a* = 9.2697 (9) Å
*b* = 16.1943 (16) Å
*c* = 8.0332 (8) Å
β = 104.395 (2)°
*V* = 1168.1 (2) Å^3^
*Z* = 2
Mo *K*α radiation
μ = 0.10 mm^−1^
*T* = 273 K
0.30 × 0.25 × 0.15 mm

#### Data collection

Bruker APEX CCD area-detector diffractometer
Absorption correction: multi-scan (*SADABS*; Sheldrick, 1997 ▶) *T*_min_ = 0.972, *T*_max_ = 0.986
6999 measured reflections
2717 independent reflections
2458 reflections with *I* > 2σ(*I*)
*R*_int_ = 0.021

#### Refinement

*R*[*F* ^2^ > 2σ(*F* ^2^)] = 0.049
*wR*(*F* ^2^) = 0.121
*S* = 1.06
2717 reflections
172 parameters
H-atom parameters constrained
Δρ_max_ = 0.37 e Å^−3^
Δρ_min_ = −0.30 e Å^−3^

### 

Data collection: *SMART* (Bruker, 2001 ▶); cell refinement: *SAINT* (Bruker, 2002 ▶); data reduction: *SAINT*; program(s) used to solve structure: *SHELXS97* (Sheldrick, 2008 ▶); program(s) used to refine structure: *SHELXL97* (Sheldrick, 2008 ▶); molecular graphics: *Mercury* (Macrae *et al.*, 2008 ▶); software used to prepare material for publication: *SHELXL97*.

## Supplementary Material

Crystal structure: contains datablock(s) I, global. DOI: 10.1107/S1600536811026924/kp2340sup1.cif

Structure factors: contains datablock(s) I. DOI: 10.1107/S1600536811026924/kp2340Isup2.hkl

Supplementary material file. DOI: 10.1107/S1600536811026924/kp2340Isup3.cml

Additional supplementary materials:  crystallographic information; 3D view; checkCIF report

## Figures and Tables

**Table 1:** Hydrogen-bond geometry (Å, °)

*D*—H⋯*A*	*D*—H	H⋯*A*	*D*⋯*A*	*D*—H⋯*A*
C7—H7⋯N12^i^	0.93	2.71	3.348 (2)	127
C17—H17⋯N3^ii^	0.93	2.71	3.580 (2)	153
C14—H14⋯N12^iii^	0.93	2.75	3.387 (2)	127

Symmetry codes: (i) 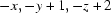; (ii) 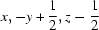; (iii) 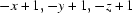.

**Fig. 1.**
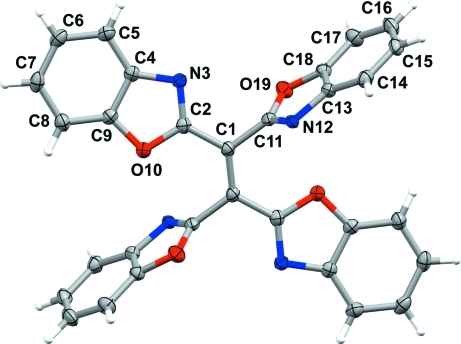
The molecular structure of the title molecule, with the atom-numbering scheme. Displacement ellipsoids are drawn at the 50% probability level. Unlabeled atoms are related to the labeled ones by the symmetry operation (i) -x,1 - y,1 - z.

**Fig. 2.**
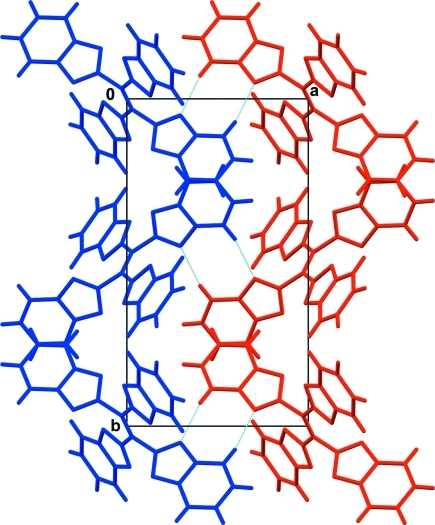
Representation of the packing viewed down the c axis. The corrugated layers formed by weak C—H···N and π–π interactions are indicated in blue and red; C14—H14···N12 contacts between these layers are shown as dotted light-blue lines.

## supplementary crystallographic information

### Comment

The title compound is reminiscent of the crystal structure of 1,1,2,2-tetrakis(1,3-benzothiazol-2-yl)ethene chloroform solvate (Hagos *et al.*, 2010) and was isolated in a similar way. The asymmetric unit consists of half of the molecule with the other part being generated by an inversion centre (Fig. 1). The conformation of the molecule resembles that of the previous compound with a dihedral angle between the planes of the benzoxazole rings attached to the same carbon of 74.39 (5)°, whereas the corresponding value in the benzothiazole derivative was 85.74 (4)°. The bond length of 1.356 (3) Å for C1—C1^i^ (symmetry operation (i): -*x*,1 - *y*,1 - *z*) is almost identical to that in the earlier described structure (1.359 (3) Å). The bond lengths for the benzoxazole rings are comparable with previously reported values (Jian *et al.*, 2007; Lokaj *et al.*, 1997; Muir *et al.*, 1992). The packing is dominated by weak C—H···N (Table 1) and π–π interactions. The hydrogen bonds between C7—H7···N12 and C17—H17···N3 generate corrugated layers which are stabilised by π-π stacking interactions of the oxazole and phenyl rings with a distance of 3.652 (1) Å between the centroids of these rings (symmetry operation: -*x*,1 - *y*,2 - *z*) and *ca.* 1.41 Å slippage. In addition to this, N12 acts as a bifurcated hydrogen bond acceptor and interlinks the layers through C14—H14···N12 weak interaction to form a three-dimensional assembly (Fig. 2).

### Experimental

A solution of bis(2-benzoxazolyl)methane (0.075 g, 0.30 mmol) in THF (20 mL) at 253 K was treated with *n*-BuLi in *n*-hexane (0.25 mL, 1.4 *M*, 0.35 mmol) and stirred for 1 h. The temperature was then slowly raised to room temperature. A suspension of an excess of S_8_ (*ca* 2 mol equivalents) in 20 mL of THF was added to the mixture at 253 K and stirred for 1 h. After treating the resulting mixture with a solution of AuCl(PPh_3_) (0.15 g, 0.33 mmol) (Bruce *et al.*, 1989) in THF (20 mL), the mixture was allowed to slowly warm to ambient temperature while stirring. The solvent was removed under reduced pressure. Crystallisation of a dichloromethane solution of the residue layered with *n*-heptane at 253 K afforded light yellow crystals. Single crystal X-ray studies revealed that the unexpected oxidative dimerisation of bis(2-benzoxazolyl)methane had occurred to yield the title compound.

### Refinement

All H atoms were positioned geometrically, with C—H = 0.93 and constrained to ride on their parent atoms with *U*_iso_(H) = 1.2*U*_eq_(C).

### Crystal data

**Table:**

C_30_H_16_N_4_O_4_	*F*(000) = 512
*M**_r_* = 496.47	*D*_x_ = 1.412 Mg m^−^^3^
Monoclinic, *P*2_1_/*c*	Mo *K*α radiation, λ = 0.71073 Å
Hall symbol: -P 2ybc	Cell parameters from 3523 reflections
*a* = 9.2697 (9) Å	θ = 2.5–28.3°
*b* = 16.1943 (16) Å	µ = 0.10 mm^−^^1^
*c* = 8.0332 (8) Å	*T* = 273 K
β = 104.395 (2)°	Block, yellow
*V* = 1168.1 (2) Å^3^	0.30 × 0.25 × 0.15 mm
*Z* = 2	

### Data collection

**Table:**

Bruker APEX CCD area-detector diffractometer	2717 independent reflections
Radiation source: fine-focus sealed tube	2458 reflections with *I* > 2σ(*I*)
graphite	*R*_int_ = 0.021
ω scans	θ_max_ = 28.3°, θ_min_ = 2.3°
Absorption correction: multi-scan (*SADABS*; Sheldrick, 1997)	*h* = −12→12
*T*_min_ = 0.972, *T*_max_ = 0.986	*k* = −17→21
6999 measured reflections	*l* = −10→7

### Refinement

**Table:**

Refinement on *F*^2^	Primary atom site location: structure-invariant direct methods
Least-squares matrix: full	Secondary atom site location: difference Fourier map
*R*[*F*^2^ > 2σ(*F*^2^)] = 0.049	Hydrogen site location: inferred from neighbouring sites
*wR*(*F*^2^) = 0.121	H-atom parameters constrained
*S* = 1.06	*w* = 1/[σ^2^(*F*_o_^2^) + (0.0513*P*)^2^ + 0.8168*P*] where *P* = (*F*_o_^2^ + 2*F*_c_^2^)/3
2717 reflections	(Δ/σ)_max_ < 0.001
172 parameters	Δρ_max_ = 0.37 e Å^−^^3^
0 restraints	Δρ_min_ = −0.30 e Å^−^^3^

### Special details

**Table:**

Geometry. All e.s.d.'s (except the e.s.d. in the dihedral angle between two l.s. planes) are estimated using the full covariance matrix. The cell e.s.d.'s are taken into account individually in the estimation of e.s.d.'s in distances, angles and torsion angles; correlations between e.s.d.'s in cell parameters are only used when they are defined by crystal symmetry. An approximate (isotropic) treatment of cell e.s.d.'s is used for estimating e.s.d.'s involving l.s. planes.
Refinement. Refinement of *F*^2^ against ALL reflections. The weighted *R*-factor *wR* and goodness of fit *S* are based on *F*^2^, conventional *R*-factors *R* are based on *F*, with *F* set to zero for negative *F*^2^. The threshold expression of *F*^2^ > σ(*F*^2^) is used only for calculating *R*-factors(gt) *etc*. and is not relevant to the choice of reflections for refinement. *R*-factors based on *F*^2^ are statistically about twice as large as those based on *F*, and *R*- factors based on ALL data will be even larger.

### Fractional atomic coordinates and isotropic or equivalent isotropic displacement parameters (Å^2^)

**Table:**

	*x*	*y*	*z*	*U*_iso_*/*U*_eq_	
C1	0.02783 (16)	0.46822 (9)	0.55330 (19)	0.0186 (3)	
C2	−0.02821 (16)	0.44239 (9)	0.69934 (19)	0.0199 (3)	
N3	0.01487 (14)	0.37588 (8)	0.78861 (16)	0.0197 (3)	
C4	−0.06473 (16)	0.37680 (9)	0.91493 (19)	0.0196 (3)	
C5	−0.06558 (18)	0.32189 (10)	1.0482 (2)	0.0240 (3)	
H5	−0.0059	0.2749	1.0664	0.029*	
C6	−0.15995 (18)	0.34088 (10)	1.1524 (2)	0.0258 (3)	
H6	−0.1640	0.3055	1.2423	0.031*	
C7	−0.24923 (18)	0.41171 (10)	1.1263 (2)	0.0262 (4)	
H7	−0.3114	0.4218	1.1988	0.031*	
C8	−0.24768 (18)	0.46738 (10)	0.9955 (2)	0.0246 (3)	
H8	−0.3060	0.5148	0.9782	0.029*	
C9	−0.15338 (16)	0.44734 (9)	0.89261 (19)	0.0199 (3)	
O10	−0.12962 (12)	0.48989 (7)	0.75253 (14)	0.0217 (3)	
C11	0.15956 (17)	0.42084 (10)	0.53413 (19)	0.0217 (3)	
N12	0.29464 (13)	0.44984 (7)	0.58136 (16)	0.0179 (3)	
C13	0.38086 (17)	0.38841 (9)	0.53122 (19)	0.0194 (3)	
C14	0.53275 (17)	0.38556 (10)	0.5437 (2)	0.0251 (3)	
H14	0.5959	0.4286	0.5920	0.030*	
C15	0.58507 (18)	0.31551 (11)	0.4807 (2)	0.0284 (4)	
H15	0.6863	0.3111	0.4872	0.034*	
C16	0.4904 (2)	0.25091 (11)	0.4074 (2)	0.0309 (4)	
H16	0.5307	0.2048	0.3666	0.037*	
C17	0.33806 (19)	0.25342 (10)	0.3935 (2)	0.0284 (4)	
H17	0.2746	0.2106	0.3450	0.034*	
C18	0.28725 (16)	0.32445 (9)	0.45730 (19)	0.0205 (3)	
O19	0.14322 (12)	0.34621 (7)	0.46062 (15)	0.0258 (3)	

### Atomic displacement parameters (Å^2^)

**Table:**

	*U*^11^	*U*^22^	*U*^33^	*U*^12^	*U*^13^	*U*^23^
C1	0.0167 (7)	0.0186 (7)	0.0206 (7)	−0.0016 (5)	0.0050 (5)	−0.0009 (5)
C2	0.0164 (7)	0.0216 (7)	0.0211 (7)	−0.0023 (5)	0.0037 (6)	−0.0013 (6)
N3	0.0192 (6)	0.0191 (6)	0.0211 (6)	−0.0002 (5)	0.0057 (5)	0.0020 (5)
C4	0.0190 (7)	0.0195 (7)	0.0197 (7)	−0.0035 (5)	0.0037 (6)	−0.0023 (5)
C5	0.0274 (8)	0.0202 (7)	0.0233 (8)	−0.0018 (6)	0.0044 (6)	0.0016 (6)
C6	0.0314 (8)	0.0260 (8)	0.0199 (7)	−0.0096 (6)	0.0062 (6)	−0.0002 (6)
C7	0.0272 (8)	0.0306 (8)	0.0236 (8)	−0.0072 (6)	0.0114 (6)	−0.0070 (6)
C8	0.0236 (8)	0.0249 (8)	0.0266 (8)	−0.0004 (6)	0.0088 (6)	−0.0039 (6)
C9	0.0202 (7)	0.0197 (7)	0.0189 (7)	−0.0033 (5)	0.0035 (5)	−0.0007 (6)
O10	0.0217 (5)	0.0216 (5)	0.0234 (5)	0.0014 (4)	0.0084 (4)	0.0019 (4)
C11	0.0239 (8)	0.0227 (7)	0.0197 (7)	0.0061 (6)	0.0080 (6)	0.0067 (6)
N12	0.0155 (6)	0.0149 (6)	0.0246 (6)	0.0013 (4)	0.0075 (5)	0.0011 (5)
C13	0.0210 (7)	0.0180 (7)	0.0202 (7)	0.0026 (5)	0.0069 (6)	0.0017 (5)
C14	0.0202 (7)	0.0283 (8)	0.0270 (8)	−0.0029 (6)	0.0062 (6)	0.0031 (6)
C15	0.0203 (8)	0.0376 (9)	0.0291 (8)	0.0079 (7)	0.0097 (6)	0.0068 (7)
C16	0.0343 (9)	0.0297 (9)	0.0308 (9)	0.0113 (7)	0.0123 (7)	−0.0028 (7)
C17	0.0311 (9)	0.0246 (8)	0.0291 (8)	−0.0005 (6)	0.0068 (7)	−0.0056 (6)
C18	0.0192 (7)	0.0223 (7)	0.0200 (7)	0.0019 (6)	0.0047 (6)	0.0012 (6)
O19	0.0220 (6)	0.0265 (6)	0.0283 (6)	0.0015 (4)	0.0051 (5)	−0.0013 (5)

### Geometric parameters (Å, °)

**Table:**

C1—C1^i^	1.356 (3)	C9—O10	1.3830 (18)
C1—C2	1.458 (2)	C11—N12	1.302 (2)
C1—C11	1.482 (2)	C11—O19	1.3370 (19)
C2—N3	1.3006 (19)	N12—C13	1.3966 (18)
C2—O10	1.3628 (18)	C13—C18	1.386 (2)
N3—C4	1.3952 (19)	C13—C14	1.387 (2)
C4—C9	1.393 (2)	C14—C15	1.379 (2)
C4—C5	1.394 (2)	C14—H14	0.9300
C5—C6	1.387 (2)	C15—C16	1.398 (3)
C5—H5	0.9300	C15—H15	0.9300
C6—C7	1.399 (2)	C16—C17	1.389 (2)
C6—H6	0.9300	C16—H16	0.9300
C7—C8	1.387 (2)	C17—C18	1.388 (2)
C7—H7	0.9300	C17—H17	0.9300
C8—C9	1.382 (2)	C18—O19	1.3874 (18)
C8—H8	0.9300		
			
C1^i^—C1—C2	124.77 (17)	C2—O10—C9	103.63 (11)
C1^i^—C1—C11	121.42 (17)	N12—C11—O19	116.73 (13)
C2—C1—C11	113.75 (12)	N12—C11—C1	122.70 (14)
N3—C2—O10	115.83 (13)	O19—C11—C1	120.53 (13)
N3—C2—C1	124.01 (14)	C11—N12—C13	103.70 (12)
O10—C2—C1	120.14 (13)	C18—C13—C14	121.47 (14)
C2—N3—C4	104.29 (12)	C18—C13—N12	108.27 (13)
C9—C4—C5	120.43 (14)	C14—C13—N12	130.25 (14)
C9—C4—N3	108.48 (13)	C15—C14—C13	116.37 (15)
C5—C4—N3	131.09 (14)	C15—C14—H14	121.8
C6—C5—C4	116.56 (15)	C13—C14—H14	121.8
C6—C5—H5	121.7	C14—C15—C16	121.96 (15)
C4—C5—H5	121.7	C14—C15—H15	119.0
C5—C6—C7	121.96 (15)	C16—C15—H15	119.0
C5—C6—H6	119.0	C17—C16—C15	122.06 (15)
C7—C6—H6	119.0	C17—C16—H16	119.0
C8—C7—C6	121.95 (15)	C15—C16—H16	119.0
C8—C7—H7	119.0	C18—C17—C16	115.22 (15)
C6—C7—H7	119.0	C18—C17—H17	122.4
C9—C8—C7	115.30 (15)	C16—C17—H17	122.4
C9—C8—H8	122.4	C13—C18—O19	107.75 (13)
C7—C8—H8	122.4	C13—C18—C17	122.92 (14)
C8—C9—O10	128.44 (14)	O19—C18—C17	129.34 (14)
C8—C9—C4	123.79 (14)	C11—O19—C18	103.54 (12)
O10—C9—C4	107.77 (13)		
			
C1^i^—C1—C2—N3	171.93 (18)	C1^i^—C1—C11—N12	75.2 (2)
C11—C1—C2—N3	−11.0 (2)	C2—C1—C11—N12	−102.04 (16)
C1^i^—C1—C2—O10	−9.6 (3)	C1^i^—C1—C11—O19	−102.5 (2)
C11—C1—C2—O10	167.47 (13)	C2—C1—C11—O19	80.33 (17)
O10—C2—N3—C4	0.47 (17)	O19—C11—N12—C13	1.25 (17)
C1—C2—N3—C4	178.99 (13)	C1—C11—N12—C13	−176.47 (13)
C2—N3—C4—C9	−0.45 (15)	C11—N12—C13—C18	−0.89 (16)
C2—N3—C4—C5	−179.84 (16)	C11—N12—C13—C14	178.08 (16)
C9—C4—C5—C6	1.1 (2)	C18—C13—C14—C15	−0.7 (2)
N3—C4—C5—C6	−179.58 (15)	N12—C13—C14—C15	−179.59 (15)
C4—C5—C6—C7	−0.4 (2)	C13—C14—C15—C16	0.3 (2)
C5—C6—C7—C8	−0.5 (2)	C14—C15—C16—C17	0.0 (3)
C6—C7—C8—C9	0.7 (2)	C15—C16—C17—C18	0.1 (3)
C7—C8—C9—O10	179.17 (14)	C14—C13—C18—O19	−178.77 (13)
C7—C8—C9—C4	0.0 (2)	N12—C13—C18—O19	0.32 (16)
C5—C4—C9—C8	−1.0 (2)	C14—C13—C18—C17	0.9 (2)
N3—C4—C9—C8	179.59 (14)	N12—C13—C18—C17	180.00 (14)
C5—C4—C9—O10	179.77 (13)	C16—C17—C18—C13	−0.6 (2)
N3—C4—C9—O10	0.30 (16)	C16—C17—C18—O19	179.05 (15)
N3—C2—O10—C9	−0.29 (16)	N12—C11—O19—C18	−1.06 (17)
C1—C2—O10—C9	−178.87 (13)	C1—C11—O19—C18	176.71 (13)
C8—C9—O10—C2	−179.27 (15)	C13—C18—O19—C11	0.38 (15)
C4—C9—O10—C2	−0.03 (15)	C17—C18—O19—C11	−179.28 (16)

Symmetry codes: (i) −*x*, −*y*+1, −*z*+1.

### Hydrogen-bond geometry (Å, °)

**Table:**

*D*—H···*A*	*D*—H	H···*A*	*D*···*A*	*D*—H···*A*
C7—H7···N12^ii^	0.93	2.71	3.348 (2)	127
C17—H17···N3^iii^	0.93	2.71	3.580 (2)	153
C14—H14···N12^iv^	0.93	2.75	3.387 (2)	127

Symmetry codes: (ii) −*x*, −*y*+1, −*z*+2; (iii) *x*, −*y*+1/2, *z*−1/2; (iv) −*x*+1, −*y*+1, −*z*+1.
